# Permafrost Peatland Initiation and Development in Late Holocene of the Northeast China

**DOI:** 10.1002/ece3.71212

**Published:** 2025-04-10

**Authors:** Rui Liu, Lin Zhao, Xiaodong Wu, Xiaofeng Cheng, Boxiong Zhang, Dongyu Yang, Jianxiang He, Shaoqiang Wu, Shuying Zang

**Affiliations:** ^1^ Heilongjiang Province Key Laboratory of Geographical Environment Monitoring and Spatial Information Service in Cold Regions Harbin Normal University Harbin China; ^2^ School of Geographical Sciences Nanjing University of Information Science and Technology Nanjing China; ^3^ Cryosphere Research Station on the Qinghai‐Tibet Plateau, Key Laboratory of Cryospheric Science and Frozen Soil Engineering Northwest Institute of eco‐Environment and Resources, Chinese Academy of Sciences Lanzhou China; ^4^ International Research Center for China‐Mongolia‐Russia Cold and Arid Regions Environment and Engineering Northwest Institute of eco‐Environment and Resources, Chinese Academy of Sciences Lanzhou China

**Keywords:** east Asian monsoon, greater Khingan Mountains, late Holocene, peatland evolution, permafrost, pollen, vegetation dynamics

## Abstract

Peatlands play an important role in the global carbon cycle. However, the initiation and development of permafrost peatlands and their responses to climate change remain unclear, hindering our understanding of the past and future of this region. In this study, we reconstructed the evolution of permafrost peatlands in the Greater Khingan Mountains (GKM) of Northeast China since 3500 cal yr BP using palynological evidence from permafrost peatland core and AMS^14^C dating. The results indicated that from 3500 to 2900 cal yr BP, the vegetation mainly composed of *Pinus*, thermophilic broad‐leaved trees, and Polypodiaceae, with a warm and wet climate constituting the peatland incubation period. From 2900 to 2250 cal yr BP, the vegetation mainly composed of *Pinus*, thermophilic broad‐leaved trees, and *Artemisia*, with a peatland initiation period characterized by a warm and humid climate. From 2250 to 1650 cal yr BP, the vegetation mainly composed of *Pinus*, *Betula*, and Polypodiaceae, with a cold and wet climate allowing peatland to flourish. From 1650 to 750 cal yr BP, the vegetation mainly composed of *Pinus* and *Artemisia*, and a dry, cold climate led to a slowdown or stagnation in peatland development. Later in this period, a warmer and wetter climate allowed the peatland to develop again, thereby completing the transition from eutrophic to mesotrophic state. Since 750 cal yr BP, the vegetation mainly composed of *Pinus* and Cyperaceae, indicating a colder and wetter climate allowing the peatland to flourish again, and peatlands began to change to oligotrophic state. Our results showed that the evolution of the GKM permafrost peatlands is mainly influenced by climate, and permafrost peatland development in the future will depend on trends in global climate.

## Graphical abstract

Pollen‐based analyses, the vegetation and climate of the Greater Khingan Mountains permafrost peatland in Northeast China were reconstructed. Cold and wet climatic environment would have facilitated the accumulation and vigorous development. Climate change and the resulting changes in the permafrost environment are the main drivers of the evolution of permafrost peatlands.

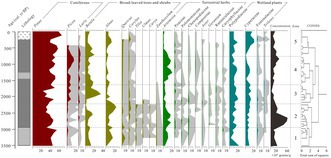

## 1 Introduction

Peatlands are important components of terrestrial ecosystems. Although peatlands occupy only approximately 3% of the Earth's land surface (Page and Baird 2016), they play a crucial role in regulating global carbon (Gorham 1991) and regional soil nutrient cycles (Aerts et al. 1999), maintaining biodiversity (Liu et al. 2022) and regulating the microclimate (Macdonald et al. 2006). Peat is a good carrier of paleoecological information because of its sensitivity to external changes, stable and continuous deposition, and ease of collection for studying climatic and environmental change (Makohonienko et al. 2008; Li et al. 2017).

Permafrost peatlands are highly sensitive to climate change as they are underlain by permafrost (Jin et al. 2019). Under cold, warm, dry, and wet climatic conditions, changes to permafrost inevitably lead to changes in peatland biology and the hydrological environment (Vardy et al. 2000). Current research indicates that mid‐high latitudes experience more pronounced ecological and environmental changes than other areas globally (Melles et al. 2019). Rapid climatic warming is also one of the greatest threats to Boreal peatlands, which are now at risk of shrinkage and degradation (Wang et al. 2022), for example, in areas such as Alaska, Northern Europe, Siberia, and Northeast China (Yu et al. 2010; Jones et al. 2012; Holmes et al. 2022).

As the most recent geological period, the Late Holocene experienced significant changes in global climate and environment (Walker et al. 2019). In turn, climate and environmental changes are also indicators of peatland evolution. Peatlands initiate and develop as a result of a combination of multiple factors, including climate, permafrost, geological landforms, and human activity (Kuhry and Turunen 2006). However, temperature and precipitation are the main factors affecting the initiation and development of permafrost peatlands (Yu et al. 2009). The climatic conditions that are necessary for the initiation and development of peatlands can usually be divided into warm and wet periods with high insolation and high precipitation, and cold and wet periods with low insolation and high precipitation (Zhao et al. 2014), both of which require the sedimentation rates (SR) of plant residues to exceed the rate of decomposition. In general, the cold and wet climate since the Late Holocene has been favorable for the development of peatlands (Tikhonravova et al. 2023).

Pollen assemblages, as “miniatures” of plants, can reliably record the features of ground flora at the time of pollen release (Bonan et al. 1992). Climatic conditions can significantly affect vegetation growth in terrestrial ecosystems (Yu et al. 2004). Because plants are highly sensitive to changes in temperature and humidity, historical vegetation succession can be an important indicator of climate and environmental change (Wang et al. 2015; Kolari et al. 2021; Gałka et al. 2018).

Northeast China is located on the southern edge of the Eurasian permafrost zone and contains a significant proportion of Boreal peatlands. These peatlands represent the largest concentrated areas of mid‐high latitude permafrost peatlands in China. Extensive research has been conducted using historical records of permafrost peatlands in northeast China, mainly concentrated on Tangbei (Yang and Wang 2002) and Youhao (Han et al. 2020) in the Lesser Khingan Mountains (LKM), Gulian (Xia 1996), Huola Basin (Zhao et al. 2016a), Beihong (Zhao et al. 2016b), Hongtu, Jintao (Gao et al. 2018), Mangui (Li et al. 2019) and Tuqiang (Han et al. 2019) in the Greater Khingan Mountains (GKM). However, these studies have primarily analyzed paleoclimatic change in peatlands, with little attention given to the evolution of these peatlands. Although Liu et al. (2024) previously analyzed the influencing factors of the evolution of permafrost peatlands in Northeast China, the initiation and development of permafrost peatlands and their response to climate change remain unclear. These knowledge gaps hinder our understanding of the past dynamics and future fate of peatlands.

Here, we present core palynological evidence extracted from permafrost peatlands in the Mohe (MH) Basin of the GKM. Based on AMS^14^C dating, principal component analysis, and detrended correspondence analysis, we attempted to reconstruct regional vegetation and climate changes since the Late Holocene, uncover the initiation and development of permafrost peatlands, compare our findings with other paleoclimatic records, and discuss possible climate forcing mechanisms.

## 2 Regional Setting

### 2.1 Study Area

**FIGURE 1:**
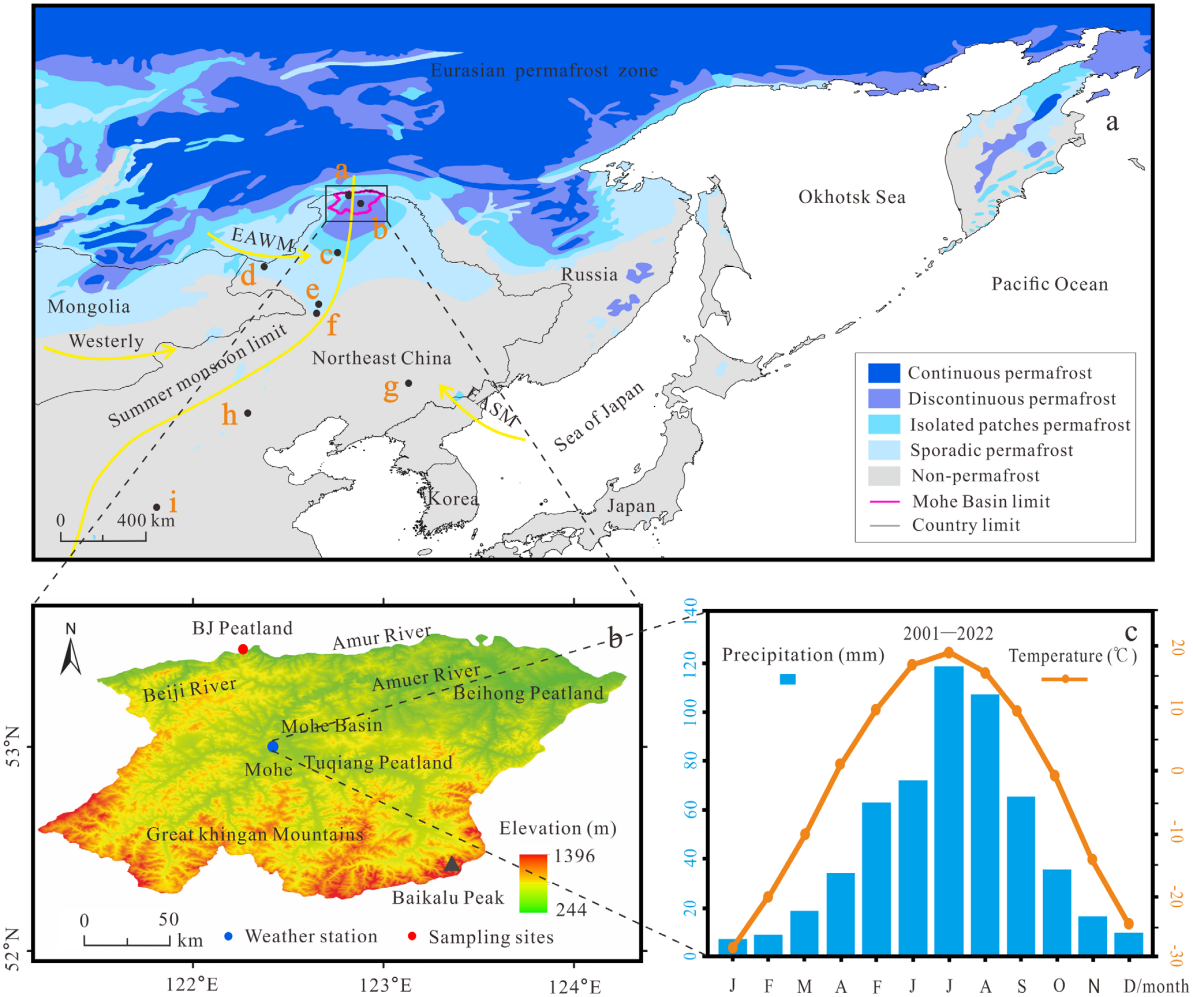
(a) Location of the BJ peatland in the Eurasian permafrost region and other sites discussed in this study ((a) BJ peatland; (b) Tuqiang peatland (Han et al. 2019); (c) Yitulihe ice core (Yang and Jin 2011); (d) Lake Hulun (Wen et al. 2010); (e) Lake Moon (Wu and Liu 2012); (f) Lake Tofengling (Sun et al. 2023); (g) Hani peatland (Hong et al. 2009); (h) Lake Daihai (Xu et al. 2010); (i) Lake Chaona (Guo et al. 2022)). The summer monsoon limit from (Gao et al. 2018) (b) The regional elevation of the MH basin. (c) Meteorological records from the Mohe weather station (data from http://data.cma.cn).

The MH Basin (52°10′–53°33′ N, 121°07′–124°20′ E Figure 1a) is the northernmost part of China. Its northern boundary is the Amur River, which connects it to the Upper Amur Basin in Russia. The periphery of the MH Basin is marked by the main ridge of the GKM, with the highest peak at Baikalu, rising to an altitude of 1396 m above sea level (a.s.l.). The surface of the MH Basin is hilly and undulating due to fluvial erosion by the Amur River and its tributaries, with a mean altitude of 450 m a.s.l. (Figure 1b).

The MH Basin represents the northern margin of the East Asian Summer Monsoon (EASM) region and is characterized by a cold temperate, continental, and monsoonal climate. Summers are warm and humid under the influence of the northwestern Pacific air mass, with precipitation concentrated in the summer months from June to September, while winters are cold and dry under the influence of the polar cold air masses/East Asian Winter Monsoon (EAWM). Mean annual precipitation (MAP) is 468.9 mm, with a mean annual temperature (MAT) of −4.9°C (Figure 1c), a monthly mean temperature of −29.7°C in January and 19.8°C in July, and a historical winter temperature extreme of −53°C (2023 ce). The surface of the MH Basin freezes for more than 8 months each year, and the mean annual ground temperature (MAGT) remains below 0°C. Belonging to typically discontinuous permafrost, a permafrost distribution area of 1.84 × 10^4^ km^2^, and an underground ice thickness of ~5 m, it is a protruding southward part of the Eurasian permafrost zone. The surface of the MH Basin is excessively wet, with a water surface depth of ~10 cm in the summer, creating large forested wetlands with a preponderance of peatlands.

### 2.2 Sampling Site

A sampling site in the Beiji Peatland (BJ Peatland, 53°28′17″ N, 122°19′47″ E, 298 m a.s.l.) in the MH Basin was chosen. The BJ Peatland soils are characterized by dark coniferous forests, swamps, and peat soils. The dominant surface species include *Betula platyphylla* , *Larix gmelinii* , *Pinus sylvestris* , *Pinus koraiensis* , and a few shrubs, including *Rhododendron dauricum* and *Betula fruticosa*, as well as wetland plants, including *Carex meyeriana*, *Polypodiodea nipponica*, and Bryophytes.

## 3 Materials and Methods

### 3.1 Peatland Core Sampling

Considering the high water content of the peatland core, which was difficult to extract, we chose the slightly frozen season in September 2017 to undertake coring. Peatland cores were extracted to a depth of 200 cm using a Russian geological drill. Because the samples were a mixture of peat and ice, to obtain sufficient samples, the core was allowed to thaw slightly, and sediment samples were continuously sliced at 5 cm intervals from the top down using a stainless steel knife. A total of 40 samples were collected; all samples were placed in labeled and sealed polyethylene (PE) plastic bags and then transferred to the laboratory for preservation in a −80°C freezer.

### 3.2 Chronological and Physicochemical Analyses

Three dating samples were selected from the BJ core: two from two different types of peat layers and one from the bottom clayey layer. They were submitted for AMS^14^C dating testing at Beta Analytics Inc. Laboratory (USA). The pH and moisture content were determined using 50 g of fresh sedimentary samples to reflect the sedimentary environmental characteristics of peat. The specific procedure involved weighing each sample on a balance, and the soil pH was directly measured using a pH tester during sampling. The samples were then placed in an aluminum specimen box to determine water content and dried in an oven at 80°C for 10 h. After drying, the samples were then removed, weighed, and the water content was calculated and expressed as percentages (%).

### 3.3 Pollen Analysis

Each 30–50 g sediment sample from the core was processed using HCl‐NaOH‐HF method. Each sample was sieved through a 10 μm mesh screen in an ultrasonic bath and mounted in glycerine. One modern *Lycopodium* spores tablet (10,315 grains/tablet) was added to each sample prior to pretreatment to calculate the pollen concentrations (grains/g) and to prepare a glass slide. Pollen identification and counting were performed using an Olympus BX‐53 light microscope at a magnification of 400×. At least 300 grains were counted in each sample (481 grains on average). Pollen counts were calculated using the absolute percentage method with the total number of pollen counts as the base. Pollen was identified using the “Chinese Plant Pollen Morphology” and “Quaternary Pollen Atlas of China” (Wang et al. 1995; Tang et al. 2016). Tilia 2.0.45 software was used for pollen diagrams and CONISS analyses (Grimm 2011).

### 3.4 Numerical Analyses

Principal component analysis (PCA) and detrended correspondence analysis (DCA) were used to investigate the relationships between pollen assemblages and environmental factors. PCA is a statistical method used to extract major gradient changes in vegetation by orthogonally transforming a set of potentially correlated variables into a set of linearly uncorrelated variables. DCA is a gradient analysis method that arranges samples or pollen taxa in a certain space and explains the trends and cyclical changes in the relationship between plant species and their environment. Fifteen major pollen genera (> 1%) from the BJ core were selected, and analyses were conducted based on square root‐transformed pollen percentage data. The gradients of the plant species and pollen assemblage bands were arranged into one or more gradients, and the results were used to reflect interrelationships between plant species and/or pollen assemblages and environmental factors (Zhao et al. 2016a, 2016b; Han et al. 2019, 2020). Origin 2022 and SPSS 20.0 were used for analysis and to extract PCA axis values.

## 4 Results

### 4.1 Lithology and Permafrost Structure

**FIGURE 2:**
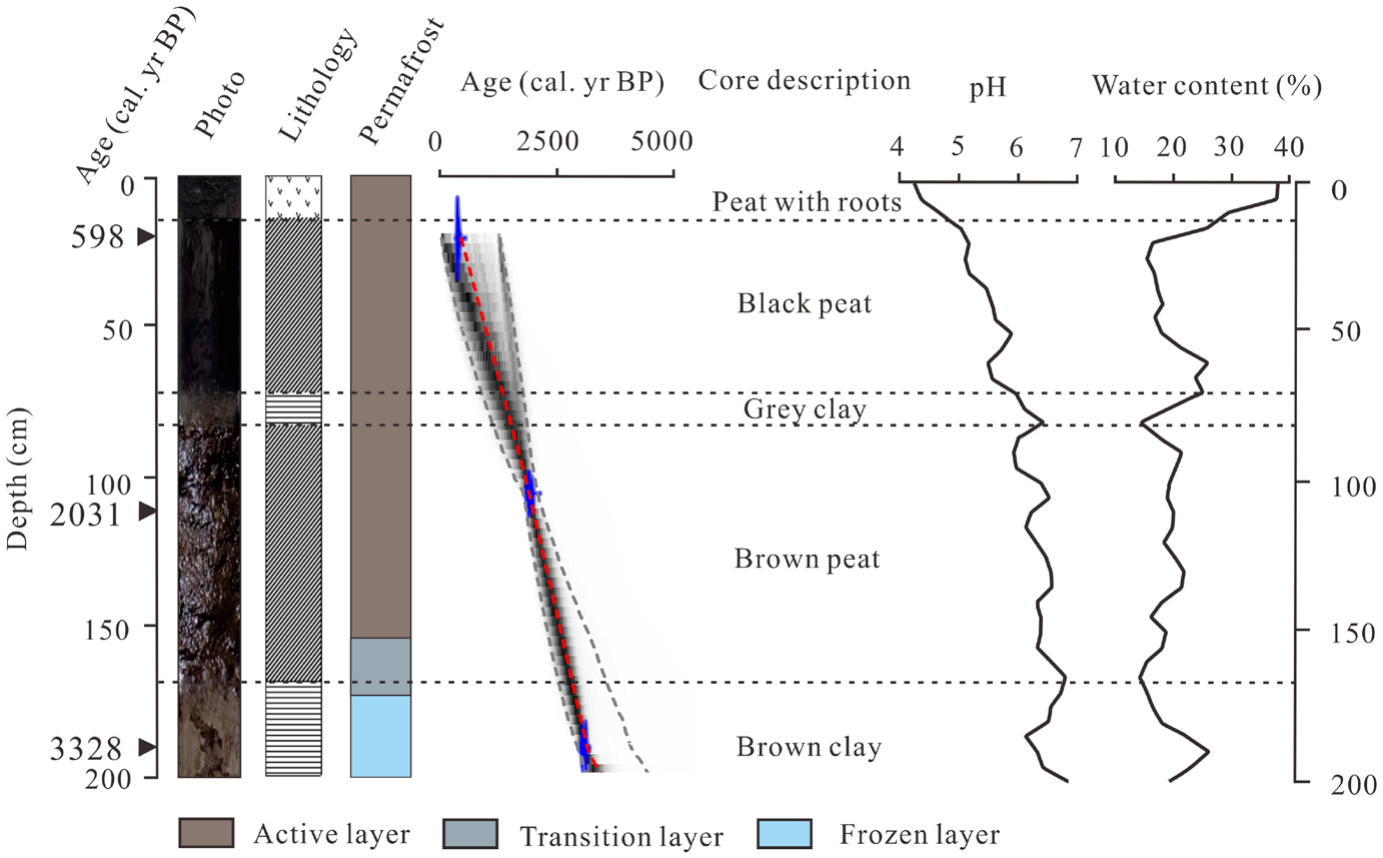
Lithology, age‐depth model, core description, pH and water content of the BJ peatland core, red dashed lines represent median age, gray dashed lines represent age range.

The lithology of the BJ core composed of five layers of alternating peat and clay from top to bottom: peat with roots (0–15 cm), black peat (15–70 cm), gray clay (70–80 cm), brown peat (80–170 cm), and brown clay (170–200 cm). The permafrost structure composed of three layers, from top to bottom: an active layer (0–160 cm), a transition layer (160–180 cm), and a frozen layer (185–200 cm) (Figure 2).

### 4.2 Chronology and Physicochemical Characteristics

The BJ core chronology based on AMS^14^C age data was established (Table 1), and a tree‐ring‐calibrated age curve (0 year BP = 1950 ce) was obtained by inputting the IntCal 20 Northern Hemisphere terrestrial standard dataset into Calib 8.1 software (Reimer et al. 2013). An age‐depth model was constructed based on the Bacon dataset in R (Blaauw and Christen 2011). The age of the bottom of the core was estimated to be 3500 cal yr BP, the age of the peat substrate was assumed to be 2900 cal yr BP, and the mean SR of the core was calculated to be ~0.57 mm/year.

**TABLE 1:** AMS^14^C radiocarbon dates for samples from the BJ peatland core.

Depth (cm)	Frozen state	Material	AMS^14^C age (year BP)	Uncertainty (±)	2σ‐range (cal yr BP)	Median age (cal yr BP)
20	Active layer	Bulk peat	600	30	582–648	589
110	Active layer	Bulk peat	2070	30	1973–2117	2031
190	Frozen layer	Bulk sediment	3110	30	3234–3391	3328

The pH of the BJ core gradually decreased from the bottom to the top of the core, with a pH range of 4.31–6.8, indicating a gradual transitional shift in peatland type. The water content of the BJ core gradually increased from the bottom to the top, fluctuating in the range of 14.43%–38.11%, with the peaty sediment at the top of the core having the highest water content. An inverse correlation was found between pH and water content (Figure 2).

### 4.3 Pollen Assemblages

**FIGURE 3:**
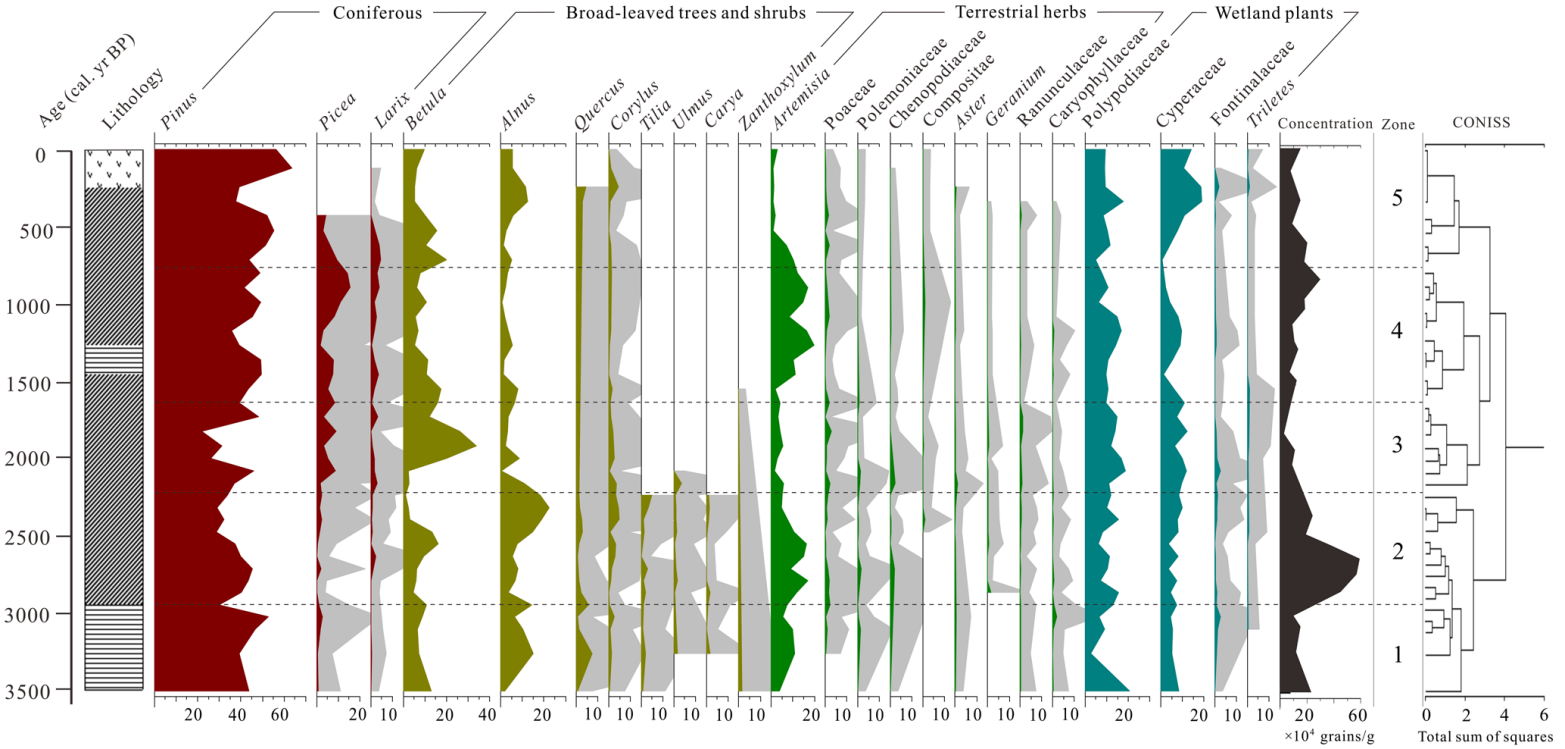
Pollen percentage diagram for the main taxa of the BJ peatland core (the gray shading is a 10× magnification of the original data).

A total of 32 pollen and spore taxa were identified in the BJ core. The pollen assemblage was dominated by trees (average 75.97%). The coniferous trees (48.5%) were mainly composed of *Pinus* (41.87%), *Picea* (4.86%), and *Larix* (1.76%). Broad‐leaved trees (27.47%), mainly *Betula* (10.17%), *Alnus* (7%), *Quercus* (2.56%), and shrubs (1.06%) had a relatively low content and were therefore grouped under broad‐leaved trees. Terrestrial herbs (16.14%) were mainly composed of *Artemisia* (7.83%), Poaceae (1.44%), and Polemoniaceae (0.72%). The wetland plants (21.28%) were mainly composed of Polypodiaceae (11.23%), Cyperaceae (7.87%), and Fontinalaceae (0.96%). Pollen concentrations ranged from 0.7 × 10^4^ to 5.6 × 10^5^ grains/g. The pollen diagram of the BJ core can be divided into five zones based on the results of the pollen CONISS analysis (Figure 3).

#### 4.3.1 Zone 1 (200–170 cm: 3500–2900 cal yr BP)

The pollen assemblage was dominated by coniferous trees, which accounted for 44.32%, mainly *Pinus* (42.33%) with a small amount of *Picea* (1.67%). Broad‐leaved trees contributed 7.72%, with *Alnus* (9.59%), *Betula* (9.22%), and *Quercus* (3.31%) as the main species. Wetland plants represented 18.23%, including Polypodiaceae (10.39%), Cyperaceae (6.14%), and Fontinalaceae (1.21%). Terrestrial herbs made up 11.33%, predominantly *Artemisia* (7.37%), Poaceae (1.02%), and Polemoniaceae (1.01%). The mean pollen concentration was 1.43 × 10^5^ grains/g, and the SR was 0.5 mm/yr.

#### 4.3.2 Zone 2 (170–120 cm: 2900–2250 cal yr BP)

Coniferous content decreased to 39.34%, mainly due to a reduction in *Pinus* (36.67%). Broad‐leaved trees content remained relatively stable at 27.05%, with an increase in *Quercus* (11.63%) and a decrease in *Betula* (6.55%). Wetland plants content remained stable at 18.86%, with a notable increase in Cyperaceae (7.05%). Terrestrial herbs content rose to 15.04%, driven by an increase in *Artemisia* (9.53%). The mean pollen concentration increased to 3.98 × 10^5^ grains/g, and the SR increased to 0.77 mm/yr.

#### 4.3.3 Zone 3 (120–80 cm: 2250–1650 cal yr BP)

Coniferous content increased to 46.8%, mainly due to an expansion of *Pinus* (38.14%) and *Picea* (6.69%). Broad‐leaved trees content decreased to 22.84%, with *Quercus* (4.19%) increasing, alongside trees and shrubs, for example, *Tilia*, *Carya*, and *Zanthoxylum*. Wetland plants content rose significantly to 22.78%, with increases in Polypodiaceae (13.18%) and Cyperaceae (8.22%). Terrestrial herbs content dropped to 7.58%, with *Artemisia* (4.23%) showing a notable decrease. The mean pollen concentration decreased to 0.87 × 10^4^ grains/g, and the SR was 0.67 mm/year.

#### 4.3.4 Zone 4 (80–30 cm: 1650–750 cal yr BP)

Coniferous content peaked at 57.11%, mainly due to the expansion of *Pinus* (45.92%) and *Picea* (8.3%). Broad‐leaved trees content significantly decreased to 13.25%, mostly composed of *Betula* (10%) and *Alnus* (2.74%). Wetland plants content decreased to 16.37%, with declines in Cyperaceae (5.1%) and Polypodiaceae (10.84%). Terrestrial herbs content increased to 13.27%, primarily driven by an increase in *Artemisia* (11.61%). The mean pollen concentration rose to 1.89 × 10^5^ grains/g, and the SR decreased to 0.5 mm/year.

#### 4.3.5 Zone 5 (30–0 cm: Since 750 cal yr BP)

Coniferous content decreased to 50.84%, but *Pinus* content continued to rise significantly, reaching its peak at 49.7%. Broad‐leaved trees content increased to 18.66%, with *Alnus* (8.17%) increasing and *Betula* (7.36%) decreasing slightly. Wetland plants content showed a marked increase to 26.63%, driven by an increase in Cyperaceae (14.61%) and a slight decrease in Polypodiaceae (10.74%). Terrestrial herbs content dropped significantly to 3.88%, with *Artemisia* (1.62%) reaching its lowest value. The mean pollen concentration decreased to 1.33 × 10^4^ grains/g, and the SR reached its minimum of 0.4 mm/year.

### 4.4 Results of PCA and DCA

PCA and DCA analyses of the 15 selected major pollen taxa revealed that the first and second principal components explained 30.5% and 15.8% of the total variance in the fossil pollen assemblages, respectively. Axis 1 had positive loadings for *Alnus*, Poaceae, *Corylus*, Fontinalaceae, and Cyperaceae, while negative loadings were observed for Polypodiaceae, *Triletes*, and *Pinus*. Axis 2 showed positive loadings for *Betula*, *Picea*, and *Artemisia*, and negative loadings for *Ulmus*, *Carya, Tilia*, and *Quercus*. Based on the PCA and DCA Axis 1 and Axis 2 scores, four clusters of corresponding plant taxa were identified, and the 38 samples were grouped into five pollen assemblage zones. Both PCA and DCA demonstrated clear relationships between different plant communities/pollen assemblage zones and between these communities and their environmental factors.

**FIGURE 4:**
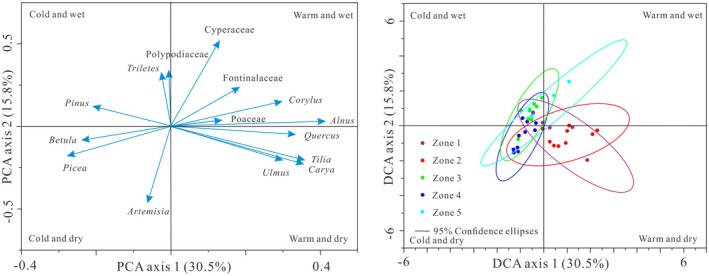
PCA and DCA results for principal pollen taxa from the BJ peatland.

**FIGURE 5:**
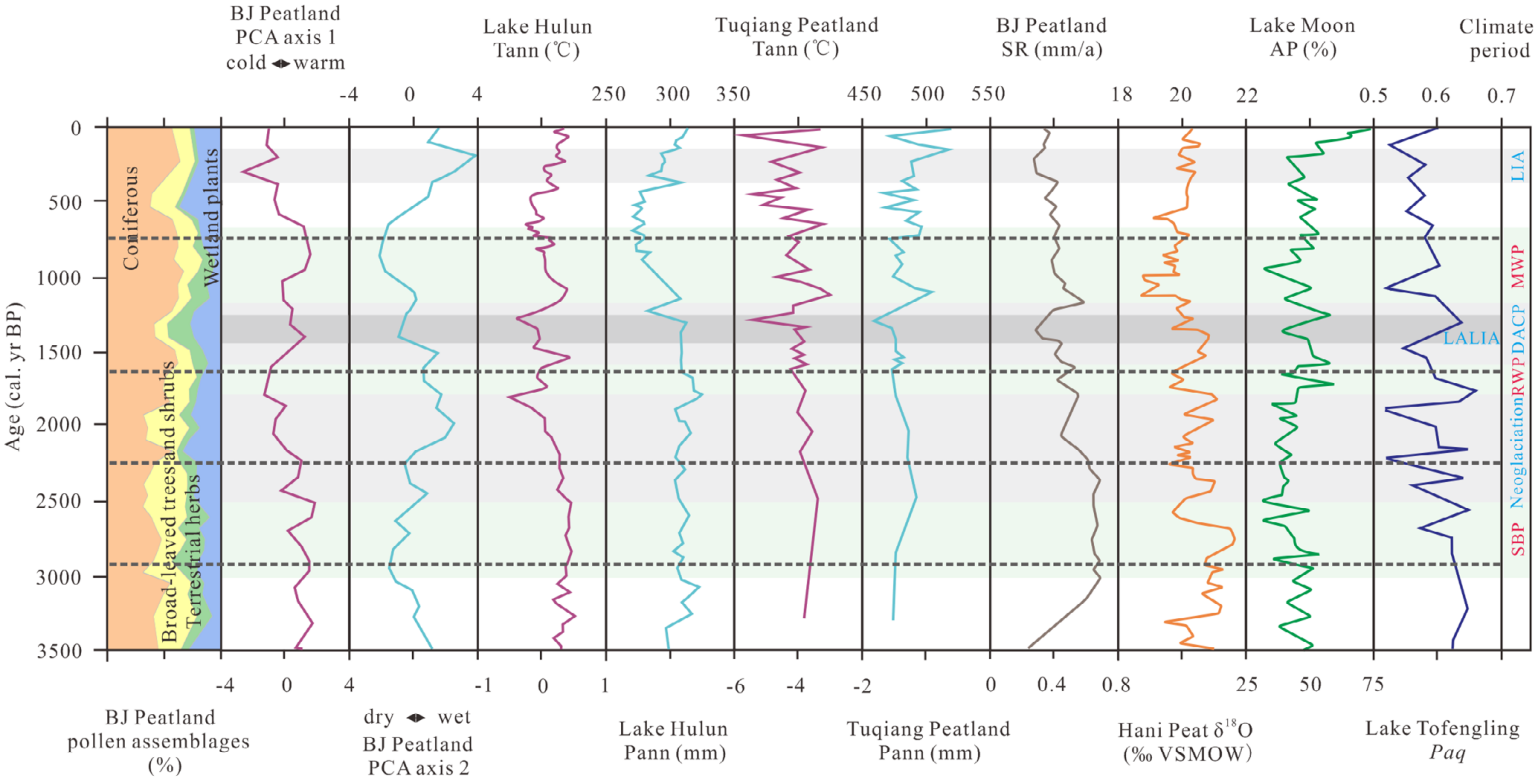
PCA axes 1 and 2 curves of the BJ peatland and comparison with other paleorecords: Lake Hulun Tann and Pann (Wen et al. 2010); the Tuqiang peatland Tann and Pann (Han et al. 2019); SR of BJ peatland (this study); δ^18^O of Hani peatland (Hong et al. 2009); AP of Lake Moon (Wu and Liu 2012); *n*‐alkanes *Paq* ratio of Lake Tofengling (Sun et al. 2023), dashed lines represent the intervals between pollen zones in the BJ peatland.

As shown in Figure 4, the Axis 1 primarily represents changes in relative temperature, with positive values indicating warmer climates and negative values indicating cooler climates. The Axis 2 reflects changes in relative moisture, with positive values indicating wetter conditions and negative values indicating drier conditions. Consequently, the Axis 1 curve provides an effective representation of plant taxa changes with temperature gradients, where lower values correspond to colder climates and higher values to warmer climates. Similarly, the Axis 2 curve effectively reflects the response of plant taxa to humidity/precipitation gradients, with lower values indicating drier conditions and higher values indicating wetter conditions (Figure 5).

## 5 Discussion

### 5.1 Reliability of the AMS^14^C Dating Results

Compared to other dating methods such as Optically Stimulated Luminescence (OSL) and ^210^Pb/^137^Cs, the AMS^14^C dating method is more suitable for peatland cores or profiles. This is primarily because peat lacks sand and quartz, which are necessary for OSL dating. Additionally, the frozen state of permafrost peat makes it difficult to ensure the absence of exposure contamination during sampling (Preusser et al. 2008). While the ^210^Pb/^137^Cs method is commonly used in peat chronology studies, its accuracy is easily affected by the sedimentary environment, and it tends to yield relatively young peat ages (Zhang et al. 2022). In contrast, the AMS^14^C method offers high accuracy for dating sediment samples up to 50,000 year and is widely employed in peat chronology research (Mokhova et al. 2009; Estop‐Aragonés et al. 2020). The principle of AMS^14^C dating is based on the fact that once an organism dies and its remains are buried in sediments, the exchange with atmospheric carbon ceases. As no new carbon is incorporated, the ^14^C content decreases at a known radioactive decay rate, allowing the age to be calculated using a half‐life of approximately 5730 year (Stuiver and Polach 1977). Peat, being an organic sediment composed of plant residues, has a high carbon content, continuous and stable sedimentation after burial, and minimal influence from groundwater “old carbon effects”. This makes peat a reliable material for dating plant death, ensuring high accuracy in peat chronology (Piotrowska et al. 2011).

The high water and ice content in the drilled core limited the number of sediment samples that could be obtained from the permafrost peatlands. Three AMS^14^C dates were obtained during the sample collection for this study. One sample was taken from the clay layer at the bottom of the peatland core, while the other two samples came from peat layers of different types. Although the bottom sample was not directly from peat, the clay in this layer contained higher organic matter, indicating it may represent a transitional stage before peat formation. Therefore, the age of this sample is also considered highly accurate. Additionally, the chronological results are consistent with the developmental ages of regional peatlands, supporting the assessment that the initiation of peatlands in the GKM of Northeast China occurred mainly during the Late Holocene (< 4000 cal yr BP) (Xing et al. 2015; Morris et al. 2018). This is reflected in the ages of other regional peatlands, including Hongtu Peatland (Gao et al. 2018), Jintao Peatland (Gao et al. 2018), Pangu Peatland (Leng 1990), Huola Peatland (Zhao et al. 2016a), Mangui Peatland (Li et al. 2019), Tuqiang Peatland (Han et al. 2019), and Gulian Peatland (Xia 1996; Table 2). In summary, based on the age‐depth model, a chronology framework was established corresponding to core depth and sedimentary layers. The chronological results showed a good linear relationship, confirming the reliability of the chronological data (Figure 2).

**TABLE 2:** Comparison of ages of peat base in the GKM permafrost peatlands.

Location	Latitude (N)	Longitude (E)	Elevation (m a.s.l.)	Sampling depth (cm)	^14^C dating quantity	Ages of peat base (cal yr BP)	References
Hongtu Peatland	51°37′12″	124°14′24″	550	59	3	750	Gao et al. 2018
Jintao Peatland	52°50′24″	123°11′24″	510	60	2	836	Gao et al. 2018
Pangu Peatland	52°41′57″	123°51′17″	589	80	2	1365	Leng 1990
Huola Basin	53°00′39″	121°57′48″	535	70	5	2000	Zhao et al. 2016a
Mangui Peatland	52°17′39″	122°08′40″	665	88	4	2110	Li et al. 2019
Tuqiang Peatland	52°56′34″	122°51′17″	481	65	4	2400	Han et al. 2019
Gulian Peatland	52°59′15″	122°03′10″	629	120	2	2435	Xia 1996
BJ Peatland	53°28′17″	122°19′47″	298	170	3	2900	This study

### 5.2 Vegetation Reflected Climate Change in BJ Peatland

The low‐lying terrain and high groundwater levels in the BJ Peatland have supported the formation of a permafrost peatland, primarily replenished by concentrated summer precipitation and the steady inflow of small tributaries from the Amur River, resulting in a mesotrophic peatland (Figure 1b). As a result, the pollen in the BJ Peatland mainly originated from the peatlands within the MH Basin and the surrounding mountains, with the pollen assemblage reflecting the ecological characteristics of these areas (Xu et al. 2016). Modern pollen studies in the GKM suggest that tree species such as *Pinus*, *Picea*, and *Betula* are indicative of a cold climate (Zhou 1991), while broad‐leaved trees such as *Quercus*, *Tilia*, *Ulmus*, *Alnus*, and *Corylus*, as well as shrubs, show a positive correlation with annual average temperature variations in Northeast China (Shen and Tang 1992; Davies and Fall 2001). Pollen genera such as *Artemisia*, Chenopodiaceae, and Poaceae dominate semi‐arid grasslands, such as the Hulunbeir Grassland in the western GKM (Wu et al. 2016). High terrestrial herb content is typically associated with a dry climate (Cui et al. 2019), while a higher abundance of wetland plants is often linked to an enhanced EASM and a wetter environment (Sun et al. 2023), as seen in genera like Cyperaceae, Polypodiaceae, Fontinalaceae, and *Triletes* (Liew et al. 2006). Based on the climate change history indicated by the vegetation in the BJ Peatland, the PCA and DCA results offer valuable insights into regional vegetation succession and climate changes over the past 3500 cal yr BP.

The interval from 3500 to 2900 cal yr BP, the dominant tree species were *Pinus*, with some *Alnus*, *Betula*, and coniferous trees being more abundant than broad‐leaved trees and shrubs. The dominant wetland plants included Polypodiaceae and Cyperaceae, while terrestrial herbs mainly consisted of small amounts of *Artemisia*. Wetland plants were more abundant than terrestrial herbs. During this period, needle and broadleaf mixed forests (with needle trees predominating) existed in the surrounding mountains, while a large area of wetland developed within the basin. The DCA and PCA results indicated that temperature and precipitation were moderate, reflecting a relatively warm and wet climate (Figure 4).

Between 2900 and 2250 cal yr BP, there was a decline in coniferous forests, primarily due to a decrease in *Pinus*, though the abundance of broad‐leaved trees and shrubs remained largely unchanged (e.g., *Carya*, *Tilia*, and *Ulmus*), in contrast to the minimal change observed in wetland plants. It is inferred that mixed coniferous broad‐leaved forests persisted in the surrounding mountains during this period, with a slight reduction in wetland areas within the basin, and some trees and terrestrial herbs began to invade the wetlands. The regional climate became slightly warmer and drier than before, as confirmed by the PCA score curve and DCA distribution range (Figure 4).

During the period of 2250 to 1650 cal yr BP, both coniferous and cold‐tolerant broad‐leaved trees (e.g., *Betula*) increased significantly, while several thermophilic tree and shrub taxa, such as *Carya*, *Tilia*, and *Zanthoxylum*, nearly disappeared. There was a marked decrease in terrestrial herbs, particularly *Artemisia*, and an increase in wetland plants, including Polypodiaceae and Cyperaceae. It is inferred that coniferous forests expanded in the surrounding mountains, and the wetland area within the basin also increased. However, the rise in *Pinus* suggests that the previously encroaching *Pinus* did not entirely retreat into the mountains. The PCA axes 1 and 2 score curves, along with the DCA distribution range, indicated that the temperature decreased, precipitation increased, and the region experienced a cold and wet climate (Figure 4).

From 1650 to 750 cal yr BP, *Pinus* and *Picea* expanded, leading to a sustained increase in coniferous taxa, reaching a maximum. There was a significant decrease in broad‐leaved trees and wetland plants, while terrestrial herbs increased. During this period, coniferous forests dominated by *Pinus* continued to spread into the surrounding mountains and basins, while herbal plants began to invade wetland areas, reducing the wetland coverage. This suggests a cold and dry climate, though between 900 and 700 cal yr BP, the climate began to warm, marked by a significant rise in broad‐leaved trees and wetland plants, indicating a shift to a warm and wet climate. The PCA and DCA results also corroborate this climatic shift (Figure 4).

Since 750 cal yr BP, *Pinus* remained the dominant species in the region, terrestrial herbs declined significantly, and wetland plants expanded rapidly, with Cyperaceae becoming the dominant group. It is concluded that coniferous forests, particularly *Pinus*, expanded in the surrounding mountains, while wetland development within the basin continued. The pollen assemblages, supported by the PCA and DCA results, indicate that the overall climate became colder and more humid (Figure 4).

### 5.3 Reconstruction of Evolutionary Process in the GKM Permafrost Peatlands

#### 5.3.1 Peatland Incubation Period (3500–2900 cal yr BP)

A warm and wet climate in the BJ Peatland is reflected by the pollen assemblages, which align with previous findings based on pollen reconstructions of annual temperature (Tann) and annual precipitation (Pann) in Lake Hulun (Wen et al. 2010). Similarly, high precipitation was recorded in the δ^18^O records of Hani Peatland (Hong et al. 2009), and high water level was indicated by the *Paq* of Lake Tuofengling (Sun et al. 2023). These results collectively support the climatic conditions of that period (Figure 5). Furthermore, the BJ Peatland pollen is rich in thermophilic broad‐leaved trees and wetland plants, which is consistent with the expansion of C_4_ plants in the GKM (Ma et al. 2018) and the occurrence of severe flooding in the plains and basins of northern Northeast China (Liu et al. 2017). In Pollen Zone 1 of the BJ core, there is a sudden increase in Fontinalaceae (Figures 3 and 5). The rapid increase in SR and the presence of silty clay in the sedimentary lithology suggest the formation of high water level wetlands in the BJ Peatland region. While this environment would have favored the development and expansion of wetlands, it would not have been conducive to the initiation of peatlands, which require weak water flow and shallow or stagnant water levels. As a result, only a few highland peat deposits began to form in the GKM (Xia 1996).

#### 5.3.2 Peatland Initiation Period (2900–2250 cal yr BP)

A warm and dry climate reflected by pollen in BJ Peatland, with reconstructed MAT in the Lake Hulun region and the Tuqiang Peatland universally increased by ~0.1°C–0.2°C and MAP universally decreased by > 20 mm (Wen et al. 2010; Han et al. 2019). The Lake Moon Arbor Pollen (AP) continued to decrease (Wu and Liu 2012), and the peat δ^18^O of Hani peatland briefly decreased (Hong et al. 2009), which also reflected the increase in temperature and the decrease in precipitation. Although climatic conditions would have remained unfavorable towards the initiation of the GKM peatlands during the Sub‐Boreal Period (SBP), due to low evaporation rates in the mid‐high latitudes, relative humidity at the surface increased (Xing et al. 2015), and high‐water levels were maintained by *Paq* of Lake Tuofengling (Sun et al. 2023; Figure 5). The warm and humid surface environment favored the vegetation. Similar to the Tuqiang Peatland, the BJ Peatland showed an expansion of thermophilic broad‐leaved trees, shrubs, and herbaceous plants, reflecting the flourishing vegetation, which further led to peaked pollen concentration and SR (Figures 3 and 5). This pattern implies that a warm and humid environment promoted the accumulation of primary biomass conducive to the formation of a stable wetland environment and the acceleration of the marsh gleization process (Ruppel et al. 2013). The conversion of buried woody and herbaceous plant residues to peat would therefore have been facilitated, and the regional peatlands initiated.

#### 5.3.3 Peatland Flourished Period (2250–1650 cal yr BP)

A cold and wet climate reflected by pollen in BJ Peatland, with reconstructed MAT for the Lake Hulun region and the Tuqiang Peatland universally decreased by ~1°C, while MAP universally increased by ~30–50 mm (Wen et al. 2010; Han et al. 2019). The peat δ^18^O records of Hani Peatland recorded (Hong et al. 2009), and high‐water level recorded by *Paq* of Lake Tuofengling (Sun et al. 2023) confirmed the cold and wet climate during this period. These findings show a close match with climatic characteristics that are consistent with the Neoglacial (Figure 5). The cold and wet climate, coupled with significant expansion of cryophilic trees and wetland plants, provides a material basis for peat accumulation, resulting in reduced decomposition of plant residues and high SR (Figures 3 and 5). These conditions favored peat development, implying that there was likely a strong period of peat development in the GKM region during this period, as also recorded in several regions of Northeast China (Yang and Wang 2003; Jiang et al. 2008). This situation persisted until the late period of ~1800 cal yr BP, gradually altered by the warm and dry climate characteristic of the Roman Warm Period (RWP).

#### 5.3.4 Peatland Slowdown or Stagnation Period (1650–750 cal yr BP)

Pollen records from BJ Peatland, Hulun Lake, and Tuqiang Peatland all reflect a cold and dry climate conditions (Wen et al. 2010; Han et al. 2019). The AP in Lake Moon suddenly decreased (Wu and Liu 2012), and the peat δ^18^O records from Hani Peatland indicate a decrease in temperature and precipitation (Hong et al. 2009). Additionally, the *Paq* from Lake Tuofengling suggests low surface water levels in the early period (Sun et al. 2023), indicating a slow recovery of temperature and precipitation in the GKM region after the end of the Neoglacial (Figure 5). These cold and dry conditions likely correspond to the Dark Ages Cold Period (DACP), which lasted from 1600 to 1200 cal yr BP. The decline in pollen concentration, particularly the significant decrease in wetland plants, reflects sparse vegetation cover, leading to slow development in BJ Peatland. During the harsh climatic conditions of the Late Antique Little Ice Age (LALIA) ~1300 to 1400 cal yr BP, the prolonged cold and dry climate resulted in a minimum relative abundance of regional vegetation (Figure 3), with an extremely low SR (~0.3 mm/year) (Figure 5). By ~900 cal yr BP, a warm and humid climate characteristic of the Medieval Warm Period (MWP) began to emerge. The increase in pollen concentrations during this time suggests the development of peatlands.

#### 5.3.5 Peatland Once Again Flourished Period (Since 750 cal yr BP)

The reconstruction of Tann and Pann revealed warm temperatures and decreased precipitation in Lake Hulun and Tuqiang Peatland. The continued decrease in AP at Lake Moon, the peat δ^18^O records from Hani Peatland, and the high‐water level maintained by *Paq* from Lake Tuofengling all reflect a dry climate (Figure 5). Furthermore, pollen‐based reconstructions of precipitation changes in the North China Plain and the Loess Plateau from Lake Daihai and Lake Chaona are consistent with these changes since the Late Holocene. In contrast, BJ Peatland reflects a cold and wet climate. This indicates differences in climate between the permafrost regions of the GKM and other EASM regions in China. Especially since the Little Ice Age (LIA), the further development of coniferous and wetland plants, represented by *Pinus* and Cyperaceae, led to the formation of a coniferous‐wetland landscape (Figures 3 and 5). These findings are in line with those of Zheng et al. (2018), who suggested that the climate in the northern part of Northeast China became colder and wetter during this period. The cold and wet climate likely promoted the rapid accumulation and expansion of regional peatlands, maintaining a high SR. This supports the hypothesis of Weckström et al. (2010) that Boreal peatlands experienced accelerated development during the Late Holocene.

### 5.4 Possible Forcing for Evolution of GKM Permafrost Peatlands

The GKM peatlands are situated in a region influenced by both the EASM and permafrost (Figure 1a), making peatland initiation and development particularly sensitive to changes in both climate and permafrost conditions. Temperature fluctuations during the Holocene were driven by orbitally induced variations in summer solar radiation in the mid‐high latitudes of the Northern Hemisphere (Wen et al. 2017). Precipitation in Northeast China is closely linked to the intensification and weakening of the EASM, as well as the dynamics of surrounding ocean–atmosphere interactions that govern the expansion of EASM‐related precipitation (An 2000). Additionally, ice core records provide insights into changes in ice volume in the Northern Hemisphere, while ice wedges offer direct evidence of permafrost expansion (Jin et al. 2019).

A comparison of peat base ages reveals that the earliest record of peatland in the EASM region dates back to the Late Pleistocene (> 40,000 cal yr BP) at Lake Dajiu in southern China (He et al. 2015). Interestingly, the earliest record of peatland in the South Asian Summer Monsoon (SASM) region of India's Nlgiri Hills coincides closely at 40,000 cal yr BP (Rajagopalan et al. 1997), reflecting synchronicity within the monsoon system. Following the Younger Dryas (YD) event (~13,000 cal yr BP), there was a peak period of peatland initiation in the EASM region of China. Similarly, peatland initiation in South Asia and Southeast Asia within the SASM region corresponded to the timing of peatland initiation in global Boreal peatlands (Morris et al. 2018). During the Early Holocene (10,000–8000 cal yr BP), the SASM region saw a peak period of peatland formation (Narayana 2024; Omar et al. 2022). In contrast, peatland in Northeast China and adjacent coastal areas responded more slowly, with rapid development occurring during the Mid to Late Holocene (6000–1000 cal yr BP). However, the current development of these peatlands, along with most peatlands in the EASM and SASM regions, has entered a phase of slowdown or stagnation. This includes peatlands such as Hani Peatland, Lake Sihailongwan in the Changbai Mountains (Zhou et al. 2010; Stebich et al. 2015), Lake Xituanpiao on the Liaodong Peninsula (Ma et al. 2021), Honghe Peatland in the Sanjiang Plain (Zhang et al. 2021), Gur Peatland in the Russian Far East (Bazarova et al. 2008), Kenbuchi Peatland in Hokkaido, Japan (Igarashi 2013), and numerous peatlands across eastern China, India, and Thailand (Treat et al. 2019).

**FIGURE 6:**
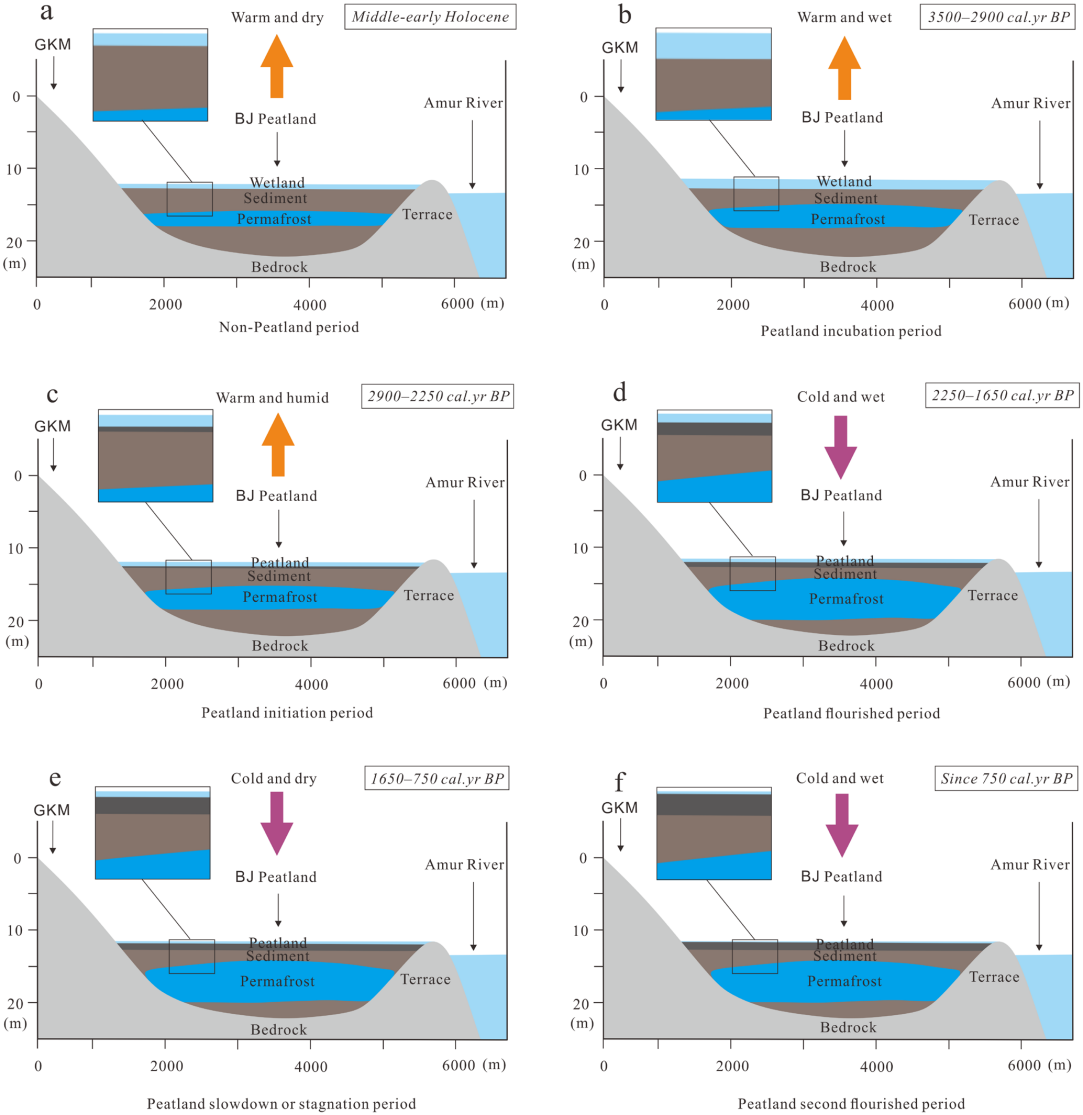
Climatic and environmental evolution of the GKM permafrost peatlands reflected by the BJ peatland; (a) Non‐Peatland period; (b) Peatland incubation period; (c) Peatland initiation period; (d) Peatland flourished period; (e) Peatland slowdown or stagnation period: (f) Peatland second flourished period.

The initiation of peatlands in the GKM occurred significantly later than in other regions of the EASM and SASM regions. Peatlands in the permafrost region of the northern part of the GKM, where BJ Peatland is located, began to form 2900 cal yr BP, with strong development following 2250 cal yr BP. While peatland development slowed in other regions, the GKM peatlands continued to expand robustly. This delay in peatland initiation in the GKM reflects a general northward delay in the development of peatlands from south to north within the EASM region of China. This was primarily due to the residual ice volumes in the Northern Hemisphere, including permafrost, mountain glaciers, and snow cover, which hindered the northward shift of the EASM rainband during the Early Holocene (Dyke 2004; Lambeck et al. 2014). Monsoon precipitation extended from South China in the Early Holocene to Northeast China by the Mid Holocene (~6000 cal yr BP) (Zhou et al. 2016). In contrast, the development of peatlands in the SASM region was less sensitive to global temperature changes due to their tropical location. In this region, peatland development was influenced not only by monsoon precipitation intensity but also by regional sea level and groundwater changes (Treat et al. 2019). Pollen‐based paleoclimatic reconstructions from lake and basin sediments in the GKM (Wu and Liu 2012; Zhao et al. 2016a) indicate that the climate during the Early to Mid Holocene was warmer and drier. Wetland expansion primarily occurred through an increase in area rather than depth, leading to enhanced terrestrialization of wetlands and rapid decomposition of plant residues, which hindered peatland initiation in the GKM permafrost region (Figure 6a). The Holocene Optimum (HO) was followed by a climatic event at 4200 cal yr BP, when abrupt climate change altered the global terrestrial climate and ecology (Wang et al. 2004), whereas the increasingly cold climate at the end of the HO during the Late Holocene (4000–3000 cal yr BP) coincided with peatland initiation.

**FIGURE 7:**
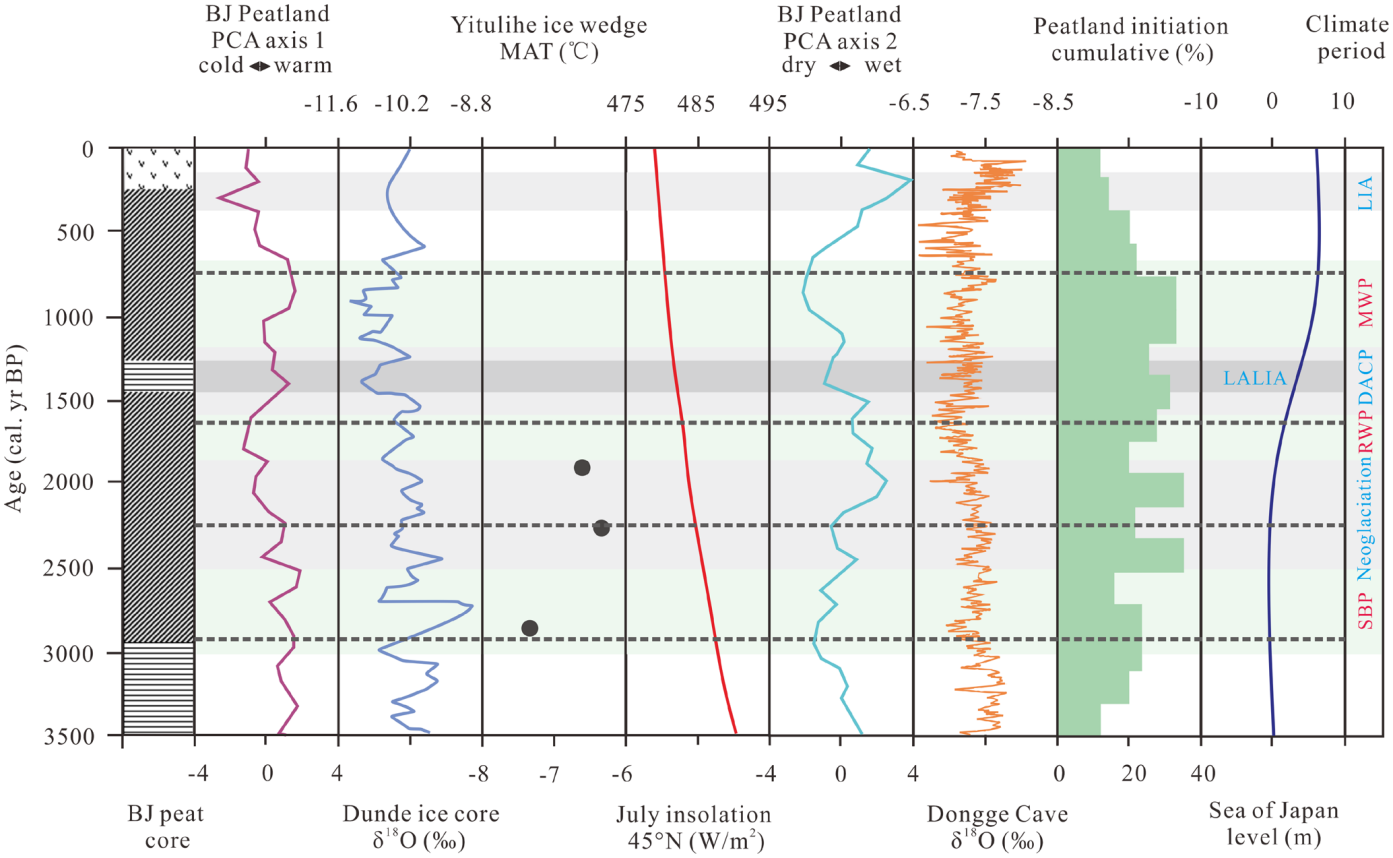
PCA axes 1 and 2 curves of the BJ peatland and correlation with potential forcings of palaeorecords: Ice core δ^18^O of the Dunde in the Tibetan Plateau (Yao and Thompson 1992); MAT of ice wedge expanded reconstructed in the Yitulihe (Yang and Jin 2011); July insolation at 45°N (Berger and Loutre 1991); stalagmite δ^18^O of Dongge Cave (Wang et al. 2005); cumulative peatland initiation and initiation frequency in Northeast China (Xing et al. 2015); sea level changes in the Sea of Japan (Spratt and Lisiecki 2016).

Since the Late Holocene, Northern Hemisphere summer insolation has decreased, but it remained relatively warm compared to the entire Late Holocene (Berger and Loutre 1991). The Dongge Cave δ^18^O records indicate a relatively strong EASM during this time (Wang et al. 2005; Figure 7). The combined effect of thawing permafrost and heavy precipitation likely led to ground oversaturation, increased surface runoff, and the expansion of lakes and wetlands in the GKM region (Zhai et al. 2011). This environment, restricting the accumulation of regional peat, rather than a high‐water level situation, would have laid the foundation for peatland initiation (Figure 6b). Based on the basal peat ages from LKM and GKM (Han et al. 2020; Xing et al. 2015), the peak period of peatland initiation occurred ~3000 cal yr BP, coinciding with the initiation of BJ Peatland. However, when a warm air mass influenced by the SBP was transported eastward by westerlies, bringing warmer and drier conditions to the mid‐high latitudes of the East Asian region (Chen et al. 2021; Figure 7), regional peatland initiation was linked to a reverse increase in surface effective humidity. This was likely due to the relatively high‐latitude location, dense vegetation cover, and the enclosed environment of the intermountain basin, which resulted in weak evaporation and maintained high effective humidity. Additionally, the δ^18^O ice core records from Dunde reflect increased ice volumes (Yao and Thompson 1992), while the expanded Yitulihe ice wedge in the GKM reconstructed a MAT of ~ − 7.3°C, similar to the conditions at the time of GKM permafrost peatland initiation (Yang and Jin 2011). The expansion of permafrost contributed to increased groundwater levels, raising surface humidity (Shur and Jorgenson 2007), while lowering the soil temperature of the active layer, which in turn slowed the decomposition of plant residues in the region (Figure 6c).

During the Neoglacial period, the rate of peat deposition in the region increased, driven by a decrease in temperature and an increase in precipitation. A cold climate was reflected in changes to the δ^18^O of ice cores from Dunde, which indicated rising ice volumes (Yao and Thompson 1992), and the expansion of the Yitulihe ice wedge in the GKM, where a reconstructed MAT of ~ − 6.4°C suggested thicker permafrost (Yang and Jin 2011; Figure 7). The presence of permafrost in the GKM region led to the retention of precipitation and surface runoff that could not infiltrate the soil. This resulted in a reducing environment with rising groundwater, promoting gleization, wetland expansion, and the accumulation of organic matter in wetland soils. This environment also increased soil fertility, enhancing vegetative productivity in both the peatland and surrounding regions (Yue et al. 2020; Figure 6d). During the RWP, temperatures increased, and precipitation decreased, primarily due to the dominance of a weakened EASM. This unfavorable climate marked the end of the period of increased regional peatlands area.

During the DACP (1600–1200 cal yr BP) and the LALIA (1400–1300 cal yr BP), the δ^18^O of Dongge Cave decreased, indicating a relatively weak EASM (Wang et al. 2005). This period saw a significant retreat in peatland development, as confirmed by reconstructed wetland records from Northeast China (Xing et al. 2015). The δ^18^O ice core records from Dunde also reflected increased ice volumes (Yao and Thompson 1992; Figure 7). The dry, frozen surface led to sparse vegetation cover, which in turn affected peat deposition (Figure 6e). In the late period (~900 cal yr BP), regional peatlands began to recover. Increased monsoon precipitation and thawing permafrost contributed to a diversification of water sources for peatland recharge. This resulted in a decrease in peat mineral content, darkening of peat color, rapid increases in water, and acidic pH (< 5.5) (Figure 2), completing the transition of the GKM peatlands from a eutrophic to a mesotrophic state.

From 750 cal yr BP to present, Northern Hemisphere summer insolation significantly decreased (Berger and Loutre 1991), while the δ^18^O record from Dongge Cave indicates an intensifying EASM (Wang et al. 2005), coupled with a rapid rise in sea level in the Sea of Japan (Spratt and Lisiecki 2016). Atmospheric cooling caused permafrost to expand once again, with the δ^18^O ice core data from Dunde reflecting increased ice volumes since the LIA (Yao and Thompson 1992; Figure 7). This cold and humid climate, along with the subsequent expansion of the permafrost layer, promoted the rapid accumulation and expansion of peatlands (Figure 6f). However, over the past century, a decrease in monsoon precipitation has persisted, while permafrost continues to maintain regional humidity and sustain the symbiotic relationship between wetlands and permafrost (Sun et al. 2011). Peatlands have also played a protective role in maintaining underlying permafrost, forming a unique type of ecologically protected permafrost (Ran et al. 2021). The developmental environment of GKM permafrost peatlands resembles that of the Siberian permafrost zone, where peatlands create cold ecosystems that interact with permafrost, vegetation, and climate (Soja et al. 2007). The current surface of the peat is strongly acidic (Figure 2), indicating that the peatlands have transitioned to an oligotrophic state. Overall, the cold and wet climate of the Late Holocene has been conducive to the initiation and development of GKM permafrost peatlands.

Although the GKM region is one of the least densely populated areas in China, human activities such as deforestation and mineral extraction have interfered with peatland development over the past 50 years, causing some small peatlands to transition into birch forests (Liu et al. 2023). Fortunately, the Chinese government has recognized the importance of permafrost peatlands and has strengthened regional ecological protection since the early 21st century. As a result, permafrost peatlands have been well‐preserved. Currently, most of these peatlands have become oligotrophic swamps (Bao 2022). The presence of mesotrophic swamps in the BJ Peatland confirms that there was a strong development in response to persistent climatic influences. The permafrost peatlands in Northeast China exhibit a MAGT close to 0°C and a MAP > 400 mm. The permafrost in this region is highly susceptible to climate change. In the context of global warming, temperatures are projected to increase by 2.4°C–4.8°C by the end of this century (Yan et al. 2007). Climate warming will not only induce the shrinkage and degradation of peatlands, but also trigger a range of ecological problems, including thawing of underlying permafrost, soil aridification, reduced biodiversity, and substantial carbon emissions. Thus, rapid climate warming poses the greatest threat to the permafrost peatlands in Northeast China.

## 6 Conclusions

Based‐Pollen evidence from the BJ Peatland core in the GKM, combined with AMS^14^C dating, PCA, and DCA methods, as well as comparisons with paleoclimatic records from other regions and globally, allowed us to summarize the initiation and development of permafrost peatlands in Northeast China since 3500 cal yr BP as follows:

From 3500 to 2900 cal yr BP, the vegetation mainly consisted of *Pinus*, thermophilic broad‐leaved trees, and Polypodiaceae. A warm and wet climate characterized this peatland incubation period. From 2900 to 2250 cal yr BP, the vegetation mainly consisted of *Pinus*, thermophilic broad‐leaved trees, and *Artemisia*. A warm and humid climate fostered peatland initiation. From 2250 to 1650 cal yr BP, the vegetation mainly consisted of *Pinus*, *Betula*, and Polypodiaceae. A cold and wet climate allowed peatland to flourish. From 1650 to 750 cal yr BP, the vegetation mainly consisted of *Pinus* and *Artemisia*. A cold and dry climate led to a slowdown or stagnation of peatland development. Later in this period, a warmer and wetter climate allowed the peatland to develop again, thereby completing the transition from eutrophic to mesotrophic state. Since 750 cal yr BP, the vegetation mainly consisted of *Pinus* and Cyperaceae. A cold and wet climate allowed the peatland to flourish again, and peatlands began to change to oligotrophic state.

The initiation and development of GKM permafrost peatlands were influenced by the delayed northward movement of the EASM. A warm and dry climate in the Early to Mid Holocene hindered peatland accumulation. Since the Late Holocene, under the coupling effect of the continuous decrease in Northern Hemisphere summer insolation, the strengthening of the EASM, and the expansion of permafrost, despite fluctuations in climate, cryophilic vegetation such as *Pinus* and Cyperaceae in the BJ Peatland would have continued to expand. The cold and wet climate facilitated the accumulation and vigorous development of GKM peatlands, leading to a rapid transformation in peatland types. Thus, climate change and the resulting changes in the permafrost environment were the primary drivers of the initiation, development, and evolution of GKM permafrost peatlands. Future developments in these peatlands will depend on global climate changes.

## Author Contributions

**Rui Liu:** data curation (lead), methodology (lead), writing – original draft (lead). **Lin Zhao:** conceptualization (lead). **Xiaodong Wu:** writing – review and editing (equal). **Xiaofeng Cheng:** software (equal), visualization (equal). **Boxiong Zhang:** software (equal), visualization (equal). **Dongyu Yang:** software (equal), visualization (equal). **Jianxiang He:** investigation (equal). **Shaoqiang Wu:** investigation (equal). **Shuying Zang:** funding acquisition (lead), project administration (lead).

## Conflicts of Interest

The authors declare no conflicts of interest.

## Supporting information


Data S1.

## Data Availability

Data available in article Supporting Information.

## References

[ece371212-bib-0001] Aerts, R. , J. T. A. Verhoeven , and D. F. Whigham . 1999. “Plant‐Mediated Controls on Nutrient Cycling in Temperate Fens and Bogs.” Ecology 80: 2170–2181. 10.1890/0012-9658(1999)080[2170:P-mconc]2.0.Co;2.

[ece371212-bib-0002] An, Z. 2000. “The History and Variability of the East Asian Paleomonsoon Climate.” Quaternary Science Reviews 19: 171–187. 10.1016/S0277-3791(99)00031-1.

[ece371212-bib-0003] Bao, K. S. 2022. Peat and Its Environmental Records in Northeast China. Science Press.

[ece371212-bib-0004] Bazarova, V. B. , M. A. Klimin , L. M. Mokhova , and L. A. Orlova . 2008. “New Pollen Records of Late Pleistocene and Holocene Changes of Environment and Climate in the Lower Amur River Basin, NE Eurasia.” Quaternary International 179, no. 1: 9–19. 10.1016/j.quaint.2007.08.015.

[ece371212-bib-0005] Berger, A. L. , and M. F. Loutre . 1991. “Insolation Values for Last 10 Million Years.” Quaternary Science Reviews 10, no. 4: 297–317. 10.1016/0277-3791(91)90033-Q.

[ece371212-bib-0006] Blaauw, M. , and J. A. Christen . 2011. “Flexible Paleoclimate Age‐Depth Models Using an Autoregressive Gamma Process.” Bayesian Analysis 6, no. 3: 457–474. 10.1214/11-BA618.

[ece371212-bib-0007] Bonan, G. B. , D. Pollard , and S. L. Thompson . 1992. “Effects of Boreal Forest Vegetation on Global Climate.” Nature 359, no. 6397: 716–718. 10.1038/359716a0.

[ece371212-bib-0008] Chen, J. , W. Huang , S. Feng , et al. 2021. “The Modulation of Westerlies‐Monsoon Interaction on Climate Over the Monsoon Boundary Zone in East Asia.” International Journal of Climatology 41, no. S1: E3049–E3064. 10.1002/joc.6903.

[ece371212-bib-0009] Cui, Q. , Y. Zhao , F. Qin , C. Liang , Q. Li , and R. Geng . 2019. “Characteristics of the Modern Pollen Assemblages From Different Vegetation Zones in Northeast China: Implications for Pollen‐Based Climate Reconstruction.” Science China Earth Sciences 62: 1564–1577. 10.1007/s11430-018-9386-9.

[ece371212-bib-0010] Davies, C. P. , and P. L. Fall . 2001. “Modern Pollen Precipitation From an Elevational Transect in Central Jordan and Its Relationship to Vegetation.” Journal of Biogeography 28, no. 10: 1195–1210. 10.1046/j.1365-2699.2001.00630.x.

[ece371212-bib-0011] Dyke, A. S. 2004. “An Outline of North American Deglaciation With Emphasis on Central and Northern Canada.” Developments in Quaternary Science 2: 373–424. 10.1016/S1571-0866(04)80209-4.

[ece371212-bib-0012] Estop‐Aragonés, C. , D. Olefeldt , B. W. Abbott , et al. 2020. “Assessing the Potential for Mobilization of Old Soil Carbon After Permafrost Thaw: A Synthesis of 14C Measurements From the Northern Permafrost Region.” Global Biogeochemical Cycles 34, no. 9: e2020GB006672. 10.1029/2020GB006672.

[ece371212-bib-0013] Gałka, M. , G. T. Swindles , M. Szal , R. Fulweber , and A. Feurdean . 2018. “Response of Plant Communities to Climate Change During the Late Holocene: Palaeoecological Insights From Peatlands in the Alaskan Arctic.” Ecological Indicators 85: 525–536. 10.1016/j.ecolind.2017.10.062.

[ece371212-bib-0014] Gao, C. , J. He , Y. Zhang , J. Cong , D. Han , and G. Wang . 2018. “Fire History and Climate Characteristics During the Last Millennium of the Great Hinggan Mountains at the Monsoon Margin in Northeastern China.” Global and Planetary Change 162: 313–320. 10.1016/j.gloplacha.2018.01.021.

[ece371212-bib-0015] Gorham, E. 1991. “Northern Peatlands: Role in the Carbon Cycle and Probable Responses to Climatic Warming.” Ecological Applications 1, no. 2: 182–195. 10.2307/1941811. 27755660

[ece371212-bib-0016] Grimm, E. C. 2011. “Tilia Software 1.7.14.” Illinois State Museum, Illinois.

[ece371212-bib-0017] Guo, C. , Y. Ma , and H. Meng . 2022. “Late Holocene Vegetation, Climate, and Lake Changes in Northern China: Varved Evidence From Western Loess Plateau.” Science of the Total Environment 827, no. 827: 154282. 10.1016/j.scitotenv.2022.154282. 35245561

[ece371212-bib-0018] Han, D. , C. Gao , H. Liu , et al. 2020. “Vegetation Dynamics and Its Response to Climate Change During the Past 2000 Years Along the Amur River Basin, Northeast China.” Ecological Indicators 117: 106577. 10.1016/j.ecolind.2020.106577.

[ece371212-bib-0019] Han, D. , C. Gao , Z. Yu , et al. 2019. “Late Holocene Vegetation and Climate Changes in the Great Hinggan Mountains, Northeast China.” Quaternary International 532: 138–145. 10.1016/j.quaint.2019.11.017.

[ece371212-bib-0020] He, Y. , C. Zhao , Z. Zheng , et al. 2015. “Peatland Evolution and Associated Environmental Changes in Central China Over the Past 40,000 Years.” Quaternary Research 84, no. 2: 255–261. 10.1016/j.yqres.2015.06.004.

[ece371212-bib-0021] Holmes, M. E. , P. M. Crill , W. C. Burnett , et al. 2022. “Carbon Accumulation, Flux, and Fate in Stordalen Mire, a Permafrost Peatland in Transition.” Global Biogeochemical Cycles 36, no. 1: e2021GB007113. 10.1029/2021GB007113.

[ece371212-bib-0022] Hong, B. , C. Liu , Q. Lin , et al. 2009. “Temperature Evolution From the δ18O Record of Hani Peat, Northeast China, in the Last 14000 Years.” Science China Earth Sciences 52, no. 7: 952–964. 10.1007/s11430-009-0086-z.

[ece371212-bib-0023] Igarashi, Y. 2013. “Holocene Vegetation and Climate on Hokkaido Island, Northern Japan.” Quaternary International 290‐291: 139–150. 10.1016/j.quaint.2012.09.030.

[ece371212-bib-0024] Jiang, W. , S. A. G. Leroy , N. Ogle , G. Chu , L. Wang , and J. Liu . 2008. “Natural and Anthropogenic Forest Fires Recorded in the Holocene Pollen Record From a Jinchuan Peat Bog, Northeastern China.” Palaeogeography Palaeoclimatology Palaeoecology 261, no. 1/2: 47–57. 10.1016/j.palaeo.2008.01.007.

[ece371212-bib-0025] Jin, H. , X. Jin , R. He , et al. 2019. “Evolution of Permafrost in China During the Last 20 Ka.” Science China Earth Sciences 62, no. 8: 1207–1223. 10.1007/s11430-018-9272-0.

[ece371212-bib-0026] Jones, M. C. , G. Grosse , B. M. Jones , and K. W. Anthony . 2012. “Peat Accumulation in Drained Thermokarst Lake Basins in Continuous, Ice‐Rich Permafrost, Northern Seward Peninsula, Alaska.” Journal of Geophysical Research: Biogeosciences 117, no. G2: 2011JG001766. 10.1029/2011JG001766.

[ece371212-bib-0027] Kolari, T. H. M. , P. Korpelainen , T. Kumpula , and T. Tahvanainen . 2021. “Accelerated Vegetation Succession but no Hydrological Change in a Boreal Fen During 20 Years of Recent Climate Change.” Ecology and Evolution 11, no. 12: 7602–7621. 10.1002/ece3.7592. 34188838 PMC8216969

[ece371212-bib-0028] Kuhry, P. , and J. Turunen . 2006. Boreal Peatland Ecosystems. Springer Press.

[ece371212-bib-0029] Lambeck, K. , H. Rouby , A. Purcell , Y. Sun , and M. Sambridge . 2014. “Sea Level and Global Ice Volumes From the Last Glacial Maximum to the Holocene.” Proceedings of the National Academy of Sciences of the United States of America 111: 15296–15303. 10.1073/pnas.1411762111. 25313072 PMC4217469

[ece371212-bib-0030] Leng, X. T. 1990. “^14^C Chronology Report in Quaternary Glaciers and Quaternary Geology Professional Committee of the Geological Society of China: Collection of Papers on Quaternary Glaciers and Quaternary Geology.” Science Press: Beijing, China.

[ece371212-bib-0031] Li, N. , F. M. Chambers , J. Yang , et al. 2017. “Records of East Asian Monsoon Activities in Northeastern China Since 15.6 ka, Based on Grain Size Analysis of Peaty Sediments in the Changbai Mountains.” Quaternary International 447: 158–169. 10.1016/j.quaint.2017.03.064.

[ece371212-bib-0032] Li, Y. Y. , B. Li , and X. Xu . 2019. “Pollen‐Based Climate Reconstruction During the Past 2100 Years From the MG Peat Profile in the Northern Daxing'an Mountains.” Quaternary Sciences 39, no. 4: 1034–1041.

[ece371212-bib-0033] Liew, P. M. , S. Y. Huang , and C. M. Kuo . 2006. “Pollen Stratigraphy, Vegetation and Environment of the Last Glacial and Holocene‐A Record From Toushe Basin, Central Taiwan.” Quaternary International 147, no. 1: 16–33. 10.1016/j.quaint.2005.09.003.

[ece371212-bib-0034] Liu, C. , X. Dong , X. Wu , et al. 2022. “Response of Carbon Emissions and the Bacterial Community to Freeze‐Thaw Cycles in a Permafrost‐Affected Forest‐Wetland Ecotone in Northeast China.” Microorganisms 10: 1950. 10.3390/microorganisms10101950. 36296226 PMC9609725

[ece371212-bib-0035] Liu, H. , Y. Cheng , O. A. Anenkhonov , et al. 2023. “Dynamics of the Climate‐Permafrost‐Vegetation Coupling System at Its Southernmost Zone in Eurasia Under Climate Warming.” Fundamental Research 9, no. 14: 114–125. 10.1016/j.fmre.2023.06.014. PMC12167863 40528952

[ece371212-bib-0036] Liu, H. , X. Yu , C. Gao , et al. 2017. “A 4000‐Yr Multi‐Proxy Record of Holocene Hydrology and Vegetation From a Peatland in the Sanjiang Plain, Northeast China.” Quaternary International 436: 28–36. 10.1016/j.quaint.2016.12.028.

[ece371212-bib-0037] Liu, R. , L. Zhao , Y. Y. Xie , L. X. Liu , S. Q. Wu , and S. Y. Zang . 2024. “Evolution of Permafrost Peatland and Its Influencing Factors in the Northern Greater Khingan Mountains Recorded by Palynology Since the Late Holocene.” Acta Ecologica Sinica 44, no. 18: 7991–8002.

[ece371212-bib-0038] Ma, R. F. , W. Zhang , P. H. Jin , et al. 2021. “Palaeo‐Vegetation and Palaea‐Climate Changes Since 13.5 Cal. ka B.P. In Jinzhou, Southern of Liaoning Province.” Quaternary Sciences 41, no. 1: 43–50.

[ece371212-bib-0039] Ma, X. Y. , Z. F. Wei , Y. L. Wang , et al. 2018. “C_3_/C_4_ Vegetation Evolution by Lake Sediments in the Huola Basin, Northeast China Since the Last Glacial Maximum.” Quaternary Sciences 38, no. 5: 1193–1202.

[ece371212-bib-0040] Macdonald, G. M. , D. W. Beilman , K. V. Kremenetski , Y. Sheng , L. C. Smith , and A. A. Velichko . 2006. “Rapid Early Development of Circumarctic Peatlands and Atmospheric CH4 and CO_2_ Variations.” Science 314, no. 5797: 285–288. 10.1126/science.1131722. 17038618

[ece371212-bib-0041] Makohonienko, M. , H. Kitagawa , T. Fujiki , X. Liu , Y. Yasuda , and H. Yin . 2008. “Late Holocene Vegetation Changes and Human Impact in the Changbai Mountains Area, Northeast China.” Quaternary International 184, no. 1: 94–108. 10.1016/j.quaint.2007.09.010.

[ece371212-bib-0042] Melles, M. , J. I. Svendsen , G. Fedorov , and B. Wagner . 2019. “Northern Eurasian Lakes‐Late Quaternary Glaciation and Climate History‐Introduction.” Boreas 48: 269–272. 10.1111/bor.12395.

[ece371212-bib-0043] Mokhova, K. , P. Tarasov , V. Bazarova , and M. Klimin . 2009. “Quantitative Biome Reconstruction Using Modern and Late Quaternary Pollen Data From the Southern Part of the Russian Far East.” Quaternary Science Reviews 28, no. 25‐26: 2913–2926. 10.1016/j.quascirev.2009.07.018.

[ece371212-bib-0044] Morris, P. J. , G. T. Swindles , P. J. Valdes , et al. 2018. “Global Peatland Initiation Driven by Regionally Asynchronous Warming.” Proceedings of the National Academy of Sciences 115, no. 19: 4851–4856. 10.1073/pnas.1717838115. PMC5948962 29666256

[ece371212-bib-0045] Narayana, A. C. 2024. “Peat Deposits of the West Coast of India: Implications for Environmental and Climate Changes During Late Quaternary.” Journal of Coastal Research 50, no. sp1: 683–687. 10.2112/JCR-SI50-129.1.

[ece371212-bib-0046] Omar, M. S. , E. Ifandi , R. S. Sukri , et al. 2022. “Peatlands in Southeast Asia: A Comprehensive Geological Review.” Earth Science Reviews 232: 104149. 10.1016/j.earscirev.2022.104149.

[ece371212-bib-0047] Page, S. E. , and A. J. Baird . 2016. “Peatlands and Global Change: Response and Resilience.” Annual Review of Environment and Resources 41: 35–57. 10.1146/annurev-environ-110615-085520.

[ece371212-bib-0048] Piotrowska, N. , M. Blaauw , D. Mauquoy , and F. M. Chambers . 2011. “Constructing Deposition Chronologies for Peat Deposits Using Radiocarbon Dating.” Mires and Peat 7, no. 10: 1–14. http://pixelrauschen.de/wbmp/media/map07/map_07_10.pdf.

[ece371212-bib-0049] Preusser, F. , D. Degering , M. Fuchs , et al. 2008. “Luminescence Dating: Basics, Methods and Applications.” E&G Quaternary Science Journal 57, no. 1/2: 95–149. 10.3285/eg.57.1-2.5.

[ece371212-bib-0050] Rajagopalan, G. , R. Sukumar , R. Ramesh , R. K. Pant , and G. Rajagopalan . 1997. “Late Quaternary Vegetational and Climatic Changes From Tropical Peats in Southern India–An Extended Record up to 40,000 Years BP.” Current Science 73, no. 1: 60–63. https://www.currentscience.ac.in/Volumes/73/01/0060.pdf.

[ece371212-bib-0051] Ran, Y. , M. T. Jorgenson , X. Li , et al. 2021. “Biophysical Permafrost Map Indicates Ecosystem Processes Dominate Permafrost Stability in the Northern Hemisphere.” Environmental Research Letters 16, no. 9: 095010. 10.1088/1748-9326/ac20f3.

[ece371212-bib-0052] Reimer, P. J. , E. Bard , A. Bayliss , et al. 2013. “Intcal 13 and Marine 13 Radiocarbon Age Calibration Curves 0‐50,000 Years Cal BP.” Radiocarbon 55, no. 4: 1869–1887. 10.2458/azu_js_rc.55.16947.

[ece371212-bib-0053] Ruppel, M. , M. Väliranta , T. Virtanen , and A. Korhola . 2013. “Postglacial Spatiotemporal Peatland Initiation and Lateral Expansion Dynamics in North America and Northern Europe.” Holocene 23, no. 11: 1596–1606. 10.1177/0959683613499053.

[ece371212-bib-0054] Shen, C. M. , and L. Y. Tang . 1992. “The Climate in the Changbai Mountain and Xiaoxing'Anling Mountain During the Holocene. The Climates and Environments of Holocene Megathermal in China.” China Ocean Press: Beijing, China.

[ece371212-bib-0055] Shur, Y. L. , and M. T. Jorgenson . 2007. “Patterns of Permafrost Formation and Degradation in Relation to Climate and Ecosystems.” Permafrost and Periglacial Processes 18, no. 1: 7–19. 10.1002/ppp.582.

[ece371212-bib-0056] Soja, A. J. , N. M. Tchebakova , N. H. F. French , et al. 2007. “Climate‐Induced Boreal Forest Change: Predictions Versus Current Observations.” Global and Planetary Change 56, no. 3/4: 274–296. 10.1016/j.gloplacha.2006.07.028.

[ece371212-bib-0057] Spratt, R. M. , and L. E. Lisiecki . 2016. “A Late Pleistocene Sea Level Stack.” Climate of the Past 12, no. 4: 1079–1092. 10.5194/cp-12-1079-2016 2016.

[ece371212-bib-0058] Stebich, M. , K. Rehfeld , F. Schlütz , P. E. Tarasov , J. Liu , and J. Mingram . 2015. “Holocene Vegetation and Climate Dynamics of NE China Based on the Pollen Record From Sihailongwan Maar Lake.” Quaternary Science Reviews 124: 275–289. 10.1016/j.quascirev.2015.07.021.

[ece371212-bib-0059] Stuiver, M. , and H. A. Polach . 1977. “Discussion Reporting of ^14^C Data.” Radiocarbon 19, no. 3: 355–363. 10.1017/S0033822200003672.

[ece371212-bib-0060] Sun, J. , X. Z. Li , X. W. Wang , J. J. Lv , Z. M. Li , and Y. M. Hu . 2011. “Latitudinal Pattern in Species Diversity and Its Response to Global Warming in Permafrost Wetlands in the Great Hing'an Mountains, China.” Russian Journal of Ecology 42, no. 2: 123–132. 10.1134/S1067413611020111.

[ece371212-bib-0061] Sun, W. , E. Zhang , E. Liu , et al. 2023. “Hydroclimate Changes Since the Last Glacial Maximum From Sedimentary Biomarkers in a Crater Lake in the Great Khingan Mountains, Northeast China.” Quaternary Science Reviews 312: 108175. 10.1016/j.quascirev.2023.108175.

[ece371212-bib-0063] Tang, L. Y. , L. M. Mao , J. W. Shu , C. H. Li , C. M. Shen , and Z. Z. Zhou . 2016. An Illustrated Handbook of Quaternary Pollen and Spores in China. Science Press.

[ece371212-bib-0064] Tikhonravova, Y. , A. Kuznetsova , E. Slagoda , and E. Koroleva . 2023. “Holocene Permafrost Peatland Evolution in Drained Lake Basins on the Pur‐Taz Interfluve, North‐Western Siberia.” Quaternary International 669: 32–42. 10.1016/j.quaint.2023.07.005.

[ece371212-bib-0065] Treat, C. C. , T. Kleinen , N. Broothaerts , V. Brovkin , and A. I. Affiliations . 2019. “Widespread Global Peatland Establishment and Persistence Over the Last 130,000 y.” Proceedings of the National Academy of Sciences of the United States of America 116, no. 11: 4822–4827. 10.1073/pnas.1813305116. 30804186 PMC6421451

[ece371212-bib-0066] Vardy, S. R. , B. G. Warner , J. Turunen , and R. Aravena . 2000. “Carbon Accumulation in Permafrost Peatlands in the Northwest Territories and Nunavut, Canada.” Holocene 10, no. 2: 273–280. 10.1191/095968300671749538.

[ece371212-bib-0067] Walker, M. , M. J. Head , J. Lowe , et al. 2019. “Subdividing the Holocene Series/Epoch: Formalization of Stages/Ages and Subseries/Subepochs, and Designation of GSSPs and Auxiliary Stratotypes.” Journal of Quaternary Science 34, no. 3: 173–186. 10.1002/jks.3097.

[ece371212-bib-0068] Wang, C. , H. Zhao , and G. Wang . 2015. “Vegetation Development and Water Level Changes in Shenjiadian Peatland in Sanjiang Plain, Northeast China.” Chinese Geographical Science 25, no. 4: 451–461. 10.1007/s11769-015-0768-8.

[ece371212-bib-0069] Wang, D. , S. Zang , L. Wang , D. Ma , and M. Li . 2022. “Effects of Permafrost Degradation on Soil Carbon and Nitrogen Cycling in Permafrost Wetlands.” Frontiers in Earth Science 10: 911314. 10.3389/feart.2022.911314.

[ece371212-bib-0070] Wang, F. X. , N. F. Qian , Y. L. Zhang , and H. Q. Yang . 1995. Pollen Flora of China. Science Press.

[ece371212-bib-0071] Wang, S. , T. Zhou , J. Cai , J. Zhu , Z. Xie , and D. Gong . 2004. “Abrupt Climate Change Around 4 Ka BP: Role of the Thermohaline Circulation as Indicated by a GCM Experiment.” Advances in Atmospheric Sciences 21: 291–295. 10.1007/BF02915716.

[ece371212-bib-0072] Wang, Y. J. , H. Cheng , R. L. Edwards , et al. 2005. “The Holocene Asian Monsoon: Links to Solar Changes and North Atlantic Climate.” Science 308, no. 5723: 854–856. 10.1126/science.1106296. 15879216

[ece371212-bib-0073] Weckström, J. , H. Seppä , and A. Korhola . 2010. “Climatic Influence on Peatland Formation and Lateral Expansion in Sub‐Arctic Fennoscandia.” Boreas 39, no. 4: 761–769. 10.1111/j.1502-3885.2010.00168.x.

[ece371212-bib-0074] Wen, R. , J. Xiao , J. Fan , S. Zhang , and H. Yamagata . 2017. “Pollen Evidence for a Mid‐Holocene East Asian Summer Monsoon Maximum in Northern China.” Quaternary Science Reviews 176: 29–35. 10.1016/j.quascirev.2017.10.008.

[ece371212-bib-0075] Wen, R. L. , J. L. Xiao , Z. G. Chang , et al. 2010. “Holocene Precipitation and Temperature Variations in the East Asian Monsoonal Margin From Pollen Data From Hulun Lake in Northeastern Inner Mongolia, China.” Boreas 39, no. 2: 262–272. 10.1111/j.1502-3885.2009.00125.x.

[ece371212-bib-0076] Wu, J. , and Q. Liu . 2012. “Pollen‐Recorded Vegetation and Climate Changes From Moon Lake Since Late Glacial.” Earth Science 37, no. 5: 947–954.

[ece371212-bib-0077] Wu, J. , Q. Liu , L. Wang , G. Q. Chu , and J. Q. Liu . 2016. “Vegetation and Climate Change During the Last Deglaciation in the Great Khingan Mountain, Northeastern China.” PLoS One 11, no. 1: e0146261. 10.1371/journal.pone.0146261. 26730966 PMC4701132

[ece371212-bib-0078] Xia, Y. 1996. “Study on Record of Spore‐Pollen in High Moor Peat and Development and Successive Process of Peat in Da and Xiao Hinggan Mountains.” Scientia Geographica Sinica 16: 337–344.

[ece371212-bib-0079] Xing, W. , K. Bao , W. Guo , X. Lu , and G. Wang . 2015. “Peatland Initiation and Carbon Dynamics in Northeast China: Links to Holocene Climate Variability.” Boreas 44, no. 3: 575–587. 10.1111/bor.12116.

[ece371212-bib-0080] Xu, Q. , F. Chen , S. Zhang , et al. 2016. “Vegetation Succession and East Asian Summer Monsoon Changes Since the Last Deglaciation Inferred From High‐Resolution Pollen Record in Gonghai Lake, Shanxi Province, China.” Holocene 27, no. 6: 835–846. 10.1177/0959683616675941.

[ece371212-bib-0081] Xu, Q. , J. Xiao , Y. Li , F. Tian , and T. Nakagawa . 2010. “Pollen‐Based Quantitative Reconstruction of Holocene Climate Changes in the Daihai Lake Area, Inner Mongolia, China.” Journal of Climate 23, no. 11: 2856–2868. 10.1175/2009JCLI3155.1.

[ece371212-bib-0082] Yan, M. , L. Liang , D. Gao , T. Li , J. Zhu , and W. Zhang . 2007. The Climate Characteristics in Northeast China: Observing Climate Change and Its Regional Difference in the Northeast China. Science Press.

[ece371212-bib-0083] Yang, S. Z. , and H. J. Jin . 2011. “δ18O and δD Records of Inactive Ice Wedge in Yitulihe, Northeastern China and Their Paleoclimatic Implications.” Science China Earth Sciences 54, no. 1: 119–126. 10.1007/s11430-010-4029-5.

[ece371212-bib-0084] Yang, Y. X. , and S. Y. Wang . 2002. “Study on Mire Development and Palaeoenvironment Change Since 9.0 Ka B.P. Inthe East Part Ofthe Xiaoxinganling Mountains.” Journal of Mountain Science 20, no. 2: 129–134.

[ece371212-bib-0085] Yang, Y. X. , and S. Y. Wang . 2003. “Study on Mire Development and Paleoenvironment Change Since 8.0 Ka B.P. Inthe Northern Part of the Sangjiang Plain.” Scientia Geographica Sinica 23, no. 1: 32–38.

[ece371212-bib-0086] Yao, T. , and L. G. Thompson . 1992. “Temperature Changes Recorded by Dunde Ice Core the Past 5ka.” Science China Earth Sciences 10: 1089–1093.

[ece371212-bib-0087] Yu, G. , X. Ke , B. Xue , and J. Ni . 2004. “The Relationships Between the Surface Arboreal Pollen and the Plants of the Vegetation in China.” Review of Palaeobotany and Palynology 129, no. 4: 187–198. 10.1016/j.revpalbo.2004.01.007.

[ece371212-bib-0088] Yu, Z. , D. W. Beilman , and M. C. Jones . 2009. “Sensitivity of Northern Peatland Carbon Dynamics to Holocene Climate Change.” Geophysical Monograph Series 184: 55–69. 10.1029/2008GM000822.

[ece371212-bib-0089] Yu, Z. , J. Loisel , D. P. Brosseau , D. W. Beilman , and S. J. Hunt . 2010. “Global Peatland Dynamics Since the Last Glacial Maximum.” Geophysical Research Letters 37, no. 13: L13402. 10.1029/2010GL043584.

[ece371212-bib-0090] Yue, Y. , H. Liu , J. Xue , Y. Li , and W. Gao . 2020. “Ecological Indicators of Near‐Surface Permafrost Habitat at the Southern Margin of the Boreal Forest in China.” Ecological Indicators 108: 105714. 10.1016/j.ecolind.2019.105714.

[ece371212-bib-0091] Zhai, D. , J. Xiao , L. Zhou , et al. 2011. “Holocene East Asian Monsoon Variation Inferred From Species Assemblage and Shell Chemistry of the Ostracodes From Hulun Lake, Inner Mongolia.” Quaternary Research 75, no. 3: 512–522. 10.1016/j.yqres.2011.02.008.

[ece371212-bib-0092] Zhang, S. , D. Wang , M. Li , F. Yan , and Q. Xu . 2022. “Significant Weak Monsoon Events During the Early to Middle Holocene Transition: Pollen Evidence From an Alpine Lake in North China.” Quaternary Science Reviews 282: 107454. 10.1016/j.quascirev.2022.107454.

[ece371212-bib-0093] Zhang, Z. , Q. Yao , Q. Xu , M. Jiang , and T. Zhu . 2021. “Hydrological and Palynological Evidence of Wetland Evolution on the Sanjiang Plain (NE China) in Response to the Holocene East Asia Summer Monsoon.” Catena 203: 105332. 10.1016/j.catena.2021.105332.

[ece371212-bib-0094] Zhao, C. , X. Li , X. Zhou , K. Zhao , and Q. Yang . 2016a. “Holocene Vegetation Succession and Responses to Climate Change in the Northern Sector of Northeast China.” Science China Earth Sciences 59: 1390–1400. 10.1007/s11430-015-5239-7.

[ece371212-bib-0095] Zhao, C. , X. Li , X. Zhou , K. Zhao , and Q. Yang . 2016b. “Holocene Vegetation Succession and Response to Climate Change on the South Bank of the Heilongjiang‐Amur River, Mohe County, Northeast China.” Advances in Meteorology 2016: 2450697. 10.1155/2016/2450697.

[ece371212-bib-0096] Zhao, Y. , Z. Yu , Y. Tang , et al. 2014. “Peatland Initiation and Carbon Accumulation in China Over the Last 50,000 Years.” Earth‐Science Reviews 128: 139–146. 10.1016/j.earscirev.2013.11.003.

[ece371212-bib-0097] Zheng, Y. , R. D. Pancost , B. D. A. Naafs , Q. Li , Z. Liu , and H. Yang . 2018. “Transition From a Warm and Dry to a Cold and Wet Climate in NE China Across the Holocene.” Earth and Planetary Science Letters 493: 36–46. 10.1016/j.epsl.2018.04.019.

[ece371212-bib-0098] Zhou, W. , Y. Zheng , P. A. Meyers , A. J. T. Jull , and S. Xie . 2010. “Postglacial Climate‐Change Record in Biomarker Lipid Compositions of the Hani Peat Sequence, Northeastern China.” Earth and Planetary Science Letters 294, no. 1/2: 37–46. 10.1016/j.epsl.2010.02.035.

[ece371212-bib-0099] Zhou, X. , L. Sun , T. Zhan , et al. 2016. “Time‐Transgressive Onset of the Holocene Optimum in the East Asian Monsoon Region.” Earth and Planetary Science Letters 456: 39–46. 10.1016/j.epsl.2016.09.052.

[ece371212-bib-0100] Zhou, Y. L. 1991. Vegetation of Da Hinggan Ling in China. China Science Press.

